# Assessment of sexual violence among street females in Bahir-Dar town, North West Ethiopia: a mixed method study

**DOI:** 10.1186/1471-2458-13-825

**Published:** 2013-09-10

**Authors:** Alemayehu C Misganaw, Yalew A Worku

**Affiliations:** 1 Clinical Trial Department, Armauer Hansen Research Institute (AHRI), Addis Ababa, Ethiopia; 2 School of Public Health, Addis Ababa University, Addis Ababa, Ethiopia

## Abstract

**Background:**

Sexual violence is a major public health concern as well as human rights violation. Homeless women are far more likely to experience violence of all sorts than women in general. The objective of this study is to assess the prevalence and consequence of rape, and explore the reasons and factors associated with rape among street females in Bahir-Dar town, North West Ethiopia.

**Methods:**

This is a mixed method study which included: a survey of 395 street females age 15 – 49; key informant interview with 4 stakeholders; 5 case studies; one focus group of 10 street females and one focus group of 10 street males. Street females are those who spend most of their time on the street and who depend on the street for their life. Qualitative and quantitative data were assessed separately with thematic and statistical analysis respectively. Quantitative data was analyzed using SPSS version 16.0. Bivariate and Multivariate analysis were determined.

**Results:**

Life time prevalence of rape was 24.3% and the prevalence of rape in the last year was 11.4%. Factors like females “off” the street [OR (95% CI) =6.2 (3.0, 12.9)], being a prostitute [OR (95% CI) = 4.1 (1.5, 11.1)] and age 15–29 [OR (95% CI) =3.5 (1.1, 11.2)] were significantly associated with rape. Most, 93.8% of the rapes were not reported to legal bodies. None of the victims used condom during the rape event. Only, 4 (4.2%) of the victims used emergency contraceptive method following the rape event. Out of the total of 96 victims of rape, 13 (19.1%) and 9 (13.2%) experienced unwanted pregnancy and induced abortion respectively. Beside, 38 (41%) and 15 (22%) victims claimed genital injury and unusual vaginal discharge respectively. Psychological consequence like, hating others by 34 (35.8%), fear and concern for HIV/AIDS by 44 (46.6%), guilt feeling by 28 (29.4%) and loss of interest in sexual activity by 28 (29.4%) of victims were reported. Majority, 42.9% of victims attribute their victimization with sleeping in areas where there are many brothels. Being physically weak, long stay in street life, and sleeping around street males were reasons mentioned for rape by 11.9%, 29.7% and 15.4% of rape victims respectively.

**Conclusion:**

In general, there is a very high prevalence of rape exacting significant physical and psychological tolls in victims of the study. Therefore, timely and integrated actions of the various stakeholders working in this area are crucial to curtail this critical human rights violation.

## Background

Sexual violence against women continues to be an increased public health problem. It takes adevastating toll on women’s lives, on their families, and on society as a whole. The Secretary-General of the United Nations (UN) claimed it as a particularly unkind human rights violation that must be eradicated [1].

Worldwide, an estimated one in three women will be physically or sexually abused; and one in five will experience rape or attempted rape in their lifetime [2]. Homeless women are far more likely to experience violence of all sorts than women in general, ranging from two to four times more likely, depending on the violence type. Approximately one homeless woman in four is homeless mainly because of her experiences with violence [3]. Lifetime risk for violent victimization including sexual assault for women who live with homelessness and mental illness is far greater [4].

Most sexual abuses continue to go unreported in societies with poorly developed social networks and intervention programs, as seen in much of Africa. Furthermore, women’s inability to negotiate safe sex and refuse unwanted sex has been linked to increased risk of reproductive health problems [5-8].

Mental and emotional distress faced by women experiencing serious abuse is overwhelming. Rape is also linked to most intractable reproductive health issues of our times including unwanted pregnancy, unsafe abortions and sexually transmitted infections. Pregnancy rates among homeless youth are much higher than the rates of the general population, and seem to increase with the instability of a youth’s housing situation. About 50% of street youth have had a pregnancy experience compared to about 33% living in shelters. Less than 10% of household youth have had a pregnancy experience [9]. Three is also high rate of HIV and sexually transmitted illnesses in the homeless youth population [10]. Almost half the women reporting serious violence also meet the criteria for major depression; one-fourth for post-traumatic stress disorder [11].

To compound the problem, most homeless females do not have access to information regarding sexual health and safety. Homeless teen mothers showed a profound lack of knowledge or interest regarding birth control and reproductive health. 50% did not believe birth control was important [12].

In Ethiopia, there were an estimated number of 150,000-200,000 street dwellers [13]. Like any part of the world, considerable numbers of street females in Ethiopia are victims of rape and some others are at risk. The prevalence of rape among street females in Addis Ababa was 15.6% and additional 20.4% attempted rape has been reported. Victims of rape were suffering from unwanted pregnancy, induced abortion, trauma of the genitalia and unusual vaginal discharge [14].

During the recent years, Bahir-Dar town has undergone rapid changes that have transformed the urban environment. The rapid increase in the number of street dwellers associated with this urbanization raise concern and warrants immediate government attention. Data regarding sexual violence associated with this increased number of homeless individuals is not available in the region. Qualitative data’s regarding perpetrates characteristics and the actual processes of rape are very scarce. Only few papers discussed sexual violence among street female dwellers [14-16] and risk factors for sexual violence among street dwellers were not conclusive. Knowing the family planning practice of victims is also crucial in designing family planning service for those who are affected.

Furthermore, this research was conducted in a very growing town, Bahir-Dar, where there is a rapid and continues influx of rural females to the town, therefore timely information on the subject of Sexual violence is indispensable to recognize and avert the perpetrators attack. So far, there was no data on this particular area in this study setting. Consequently, only little information was available to design street sensitive policies and programs.

Taking into account the aforementioned results, this method mix research ascertained the prevalence and consequence of rape among street females in Bahir-Dar town. The study also explored the reasons and factors associated with rape.

## Methods

This is a mixed method study involving both quantitative and qualitative study designs. The study was conducted in August, 2010 among street females of age 15–49 in Bahir-Dar town, North West Ethiopia. The city is one of rapidly growing cities, located 564 kms North West of Addis Ababa (the capital city of Ethiopia).

### Study locations

Prior to the commencement of data collection, the following activities were performed in association with the city workers and social affair office.

1 Identification of locations where street females were concentrated.

2 Profiling of the concentration locations and collection of quantitative information in location where street females were located.

Subsequently, 13 churches, 1 Mosque, 1 market area, the city main high way and city bus station were identified.

### Method of quantitative study

#### Participants

All street females within the age range of 15–49 years in Bahir Dar town were participated. Street beggars, vendors, jobless and street females prostitutes were included in the study. Individuals who were deaf and/or having psychiatric problem, and those who had speech disability were excluded.

#### Sample size and sampling procedure

Sample size for the quantitative study was calculated based on single proportion formula by assuming 50% prevalence and 5% marginal error to yield maximum sample size. Considering non response rate of 5%, the sample size became 404. All street females of age 15–49 who were available during the data collection period were included in the study.

Regarding the sampling strategy, a special sampling technique was applied to collect data according to the nature of the study subjects. All available beggars from13 churches, 1 mosque, market area, bus station and the main highways were taken consecutively in one day (Sunday for its convenience). Similarly all consecutive vendor street females who were available on the next day were considered in the study.

#### Data collection instrument and data collection technique

The questionnaire was developed from DHS (demographic health survey) and the US national violence against woman survey tools and it was tailored to suit the local context. The questionnaire used in the survey component of the study incorporated measures of socio-demographic position, the occurrence and reasons of rape, consequences associated with rape, disclosure and help-seeking following experiences of violence.

Data was collected through face to face interview. Sunday was collected for its convenience to collect data from all consecutive beggars. On the next day, data from all available vendor street females was collected. Snow ball sampling technique was used to identify available jobless females and street prostitutes. The data was collected through sixteen data collection teams (each team contains one female data collector and one data collection coordinator) which were evenly distributed to data collection sites. To avoid information contamination, most of the data (data from beggars and vendors) were collected in two days duration.

#### Data management and analysis

Data was entered and cleaned in a data format in EPI info version 3.5.2. Then, data was transported to SPSS version 16.0. An internal comparison was made to assess factors associated with rape. The adequacy of the sample to run the association was checked. Proportion, percentage, ratios, frequency distribution, measure of central tendency and measure of dispersion were used to describe data. Bivariate analysis was used to examine association between dependent and independent variables and multivariate logistic regression was applied to control for confounder variables. CI which contains 1 was considered statistically significant.

### Method of qualitative study

#### Sample size and sampling strategy

The qualitative study included a total of 29 participants; two male judges working in the court; one female police officer (females and children protection officer); a woman from the office of women affairs office; five victim street females for case study and 20 participants (10 street females and 10 street males) for group discussion. The minimum age of the participants was 17 year. Participants in the qualitative study were purposively selected. Forum for street children helped us to select participants for key informant interview, case study and focus group discussion. Selection of cases was made based on the result from the quantitative study.

#### Data collection instrument and data collection technique

Pertaining to the qualitative study instruments, different semi structured guides were developed and applied for focus group discussions, key-informant interview and case studies. Data were collected on reasons, disclosure and process of rape, and the characteristics of males who commit rape. The guides were translated in to Amharic language (the local language) to suit the respondents. Focus group discussion among street dwellers, key-informant interviews among stakeholders and in-depth interview among victim females were applied to collect data. Data was collected by primary investigator.

#### Data management and analysis

All qualitative data’s were audio taped and labeled for subsequent storage and transcription. Identifiable information was not included on the tape. During the fieldwork, I reviewed all qualitative data while I was collecting it and were summarized and clarified to participants for validation. Transcribed data was translated into English for analysis. Content analysis was done for summarizing the main themes from the collected data.

### Study measures

#### Street females

Females on the street, who spend most of their time on the street and who depend on the street for their life.

#### Females “off” the street

Females who depend on the street for their life and sleep on the street.

#### Female “on” the street

Females who depend on the street for their subsistence, but usually return home at night.

#### Rape

Any non-consensual of penile penetration of the vagina whether physical force is used or not, and in this study ascertained by having non-consensual sex by the respondents at any time in their life.

#### Attempted rape

It is an attempt to have non–consensual intercourse but it does not end with penile penetration on the women, and ascertained with having been exposed to any unwilling sex attempt by the respondents at any time in their life.

#### Consequences of rape

Risks contracted secondary to rape and it include tangible and psychological risks.

Ethical approval for the study was obtained from University of Gondar and Addis Continental Institute of Public Health. As rape is very much a sensitive issue, only very well trained female data collectors were engaged. Rape victims were linked to Family guidance association of Ethiopia for any counseling or medical service. Verbal consent was obtained. Confidentiality and privacy were highly maintained. To make the data collection secured, data collection coordinators were kept in place. Participants were also given a compensatory fee and a chance to seek information and referral service according to the need of the participants.

## Results

### Socio-demographic feature

From a total of 406 eligible street females identified during data the collection period, data from 395 street females were considered for analysis yielding a response rate of 97.3%. The mean age of study subjects was 27.5 with ± 9.49 SD. Most, 74.7% of participants were from 15–29 years of age group. Majority, 253(64.1) were not married and only 25(6.3) had attended formal education. About, 72 (18.2%) of the respondents experianced substances use and around one quarter of participants, 91 (23%) of them had different kinds of disabilities (Table 1).

**Table 1:** Socio-demographic and reproductive characteristics of street females in Bahir- Dar, August, 2010

**Variables**	**Category**	**No**	**Percent**
Age in years	15-29	295	74.68
	30-39	73	18.48
	40-49	27	6.84
	Mean ± SD = 27.48 ± 9.49		
Marital status	Ever married	142	35.9
	Single	253	64.1
Educational status	Illiterate	370	93.7
	Attend formal education	25	6.3
Occupation	Begging	153	38.7
	Vending	121	30.6
	Prostitution	36	9.1
	No occupation	85	21.5
Type of street females	Females ‘on’ the street	155	39.2
	Females ‘off’ the street	240	60.8
Do you use substances*	Yes	72	18.2
	No	323	81.8
Disability**	Absent	304	77
	Present	91	23
Ever had sexual intercourse	Yes	298	75.4
	No	97	24.6
Ever heard emergency contraception	Yes	90	22.5
	No	305	77.5
Comprehensive knowledge on emergency contraception	Yes	48	12
	No	347	88
Ever heard condom	Yes	270	68.4
	No	125	31.6
Importance of condom (270)	Prevent both STIs and unwanted pregnancy	31	11.5
	Prevent STIs	195	72.2
	Prevent unwanted pregnancy	44	16.3

*Substances use: Substances in this case included, chat, benzene and cigarette smoking.

**Disability: This term applies to all street females who had visual and physical/mobility impairment.

### Participants for qualitative section

The qualitative section which involved a total of 29 participants included: two male judges working in the court; one female police officer (females and children protection officer); a woman from the office of women affairs office; five victim street females for case study and 20 participants (10 street females and 10 street males) for group discussion.

### Sexual and reproductive behavior

Almost three fourth (75.4%) of the respondents were sexually active. The median age at which they started sexual activity was 17 with mean age of 17.7. Out of the total females who started sex, 50 (16.8%) of sex initiation was attributed to rape. Most, 48(12%) of the study subjects didn’t have comprehensive knowledge on emergency contraception (Table 1).

### Prevalence of rape

A total of 96 (24.3%) street females were raped in their life time. Slightly higher than half of rape victims, 50(52.63%) were attacked once in their life time. Fewer than half, 46(47.39) were repeatedly raped ranging from 2–4 to greater than 5 times accounting 42(43.16%) and 4(4.21%) respectively. The life time number of attempted rape was 64 making its prevalence 16.2%. In general, a total of 162 life time rape episodes were reported by the study subjects making the average number of rape attack per individual 1.69. The prevalence of rape in the last year was 45 (11.4%). Four (1%) of females were raped while they were pregnant.

A street girl who was 18 years old said that, “*during the night while I was sleeping, a man hugged my neck forcibly and raped me*.”

A 19 years old physically disabled Street female reported, *“many street boys raped me countless times.*”

### Disclosure of rape events

Out of the total of 96 victims, 25 (26.05%) of them disclosed their case to someone else including legal bodies. The rest, 71(73.95%) didn’t report to anyone else. Among 25 victims who disclosed their case, only, 6 (24%) reported it to legal bodies (police and court). Majority, 14(56%) have reported to their friends only. The remaining, 4(16%) and 1(4%) reported it to their relative and street leaders respectively. The dominant reason not for reporting was embarrassment by 19(29.7%) of victims. Other reasons stated were fear of rejection by legal bodies, lack of awareness where to report, fear of retribution and concern for children by 25%, 20%, 23% and 3.2% of victims respectively.

“Many street females who were victims of rape and who cried many times had no one to listen and support them. That is why I did not report my case anywhere”.

“I didn’t tell my story for anyone because I know no one could accept my compliant”.

Both participants from the in-depth interview and FGD claimed that low awareness by the victims, fear of stigma and subsequent street life, lack of full evidence on their case hindered most victims from reporting to legal bodies.

### Perpetrates of rape

From the total 96 victims of rape, more than half, 51 (53.12%) of the victims didn’t know the person who raped them. About, 22 (22.9%) of victims were raped by their intimate partner. The rest victims were perpetrated by other known person, their relatives and state authority with 11 (11.45%), 8 (8.33%) and 4(4.16%) respectively.

Many of the rape attacks were committed by drunken males. The 19 years old street female said, “a driver man who drunk and whom I saw many times before raped her.”

A 24 years old victim female whom I talked with said *“it was at night while I was sleeping with my friend, two men whom we didn’t know before came and asked us for sexual intercourse. We refused them but using force they moved us separately to other hidden site where they raped us*”.

Most interviewees and discussants including those from the legal bodies reported that majority of the rapists were strange to victims of rape. They also claimed that some of the rapists were males that victims know before. One of the female discussant said *“frequent interaction is perceived as love by the male and the male take sexual revenge even when the female rejects his request*”.

They also said rapists have no strict age and professional boundary; therefore, it is difficult to conclude the kind of males who commit rape as you could find conscious males who attack females deliberately by breaking their houses. One of the judge told me a story, *“there are males who are out of the normal sexual desire called sesegna,”* he said *“there was a 60 years old man who was accused of for the rape act that he execute from a child he cared and there were other many cases who were arrested and accused of while committing the same crime repeatedly”.*

### Consequences of rape

Pertaining to consequences of rape, 28(29.2%) claimed that there was nothing they had experienced secondary to the rape attack, on the other hand, 68(70.8%) reported various kinds of physical and psychological of consequences. Trauma of genitalia by 38 (41%), unwanted pregnancy 13(19.1%), and unusual vaginal discharge by 15 (22%) and induced abortion by 9 (13.2%) were the major physical consequence’s whereas hating others by 34(35.8%) and fear and concern for HIV/AIDS by 44 (46.6%) were the most psychological consequences reported by victims (Table 2).

**Table 2:** Consequences and grounds of rape among raped street females in Bahir-Dar, August, 2010

**Variables**	**No**	**%**
Consequences (n=68)		
Physical consequences (n=68)*		
Unwanted pregnancy	13	19.1
Induced abortion	9	13.2
Trauma of Genitalia	38	41
Unusual discharge	15	22
Total	**75**	100
Psychological consequences (n=68)*		
Continuous headache	15	***15.7***
Fear and concern for HIV/AIDS	44	46.2
Loss of interest on sexual activity	28	29.4
Guilty feeling	28	29.4
Hate others	34	35.7
Total	**149**	156.8
Reasons (n=84)**		
Sleeping in villages where abundant alcohols are served	36	42.9
Working during the night time and sleeping around street males	10	11.9
Physically weak	25	29.7
Long stay in the street.	13	15.4
Total	84	100

*multiple response.

**Response rate for this question was 87.5%.

A girl who was raped with her friend while they were sleeping together said*, “the next day when I get my friend, she couldn’t walk as a result of the attack and because she couldn’t move here and there I took her to her parents in her former residence.”* Desperately, *she continued “currently, I am raising a child with no father”.*

A 17 years girl who was victim of forceful rape attack said “*I had vaginal discharge and have a fear of acquiring HIV/AIDS”.*

### Reasons for rape

When we look at the explanations for street females who got raped, many streets victims, 36(43%) attributed it to sleeping in areas where abundant alcohols were served followed by being physically weak in 29.7% of the victims (Table 2).

Females who can’t react physically are at greater risk of rape. The 19 years old street female who has physical disability said “*I am disabled to protect myself from repeated rape attacks*”.

Females who commit sex in exchange for money are also victims of rape. *“I was very hungry when someone came to me and asked me for sex. He convinced me to have sexual intercourse by giving some amount of money. However, the guy sexes me without using condom and refused to give the money he promised before*”.

Interviewees and discussants said, male dominance, alchohol usage, drug addiction increase the chance of female victimization. Majority of female discussants reported that males attacked them since they are physically weak and don’t have a separate shelter to sleep.

Male’s dominant character was reflected frequently during males’ focus group discussion. One discussant from the male group pointed that "*rape is not illegal if the female is invited and agreed to drink because it is clear that the male is expending his money to have sex with her*".

### Practice of condom and emergency contraception

None of the victims had used condom nor had condom at their hand during the rape event. Only, 4(4.16%) victims made use of emergency contraceptive method following the rape event. Others were dissuaded from using emergency contraceptive methods for reasons such as reluctance in 38(41.76%), fear of side effects in 18(19.8%), inability to identify a clinic that offered appropriate services in 12%, lack of money in 10.9%, lack of awareness in 10.9%, and fear of stigma in 7.8% of victim participants.

### Factors associated with rape

During Bivariate analysis, variables like substance use, disability, occupation, former residence, age, marital status, type of street, and duration of street life were significant associated with rape. Variables which were significantly associated in the first model were taken and analyzed by multivariate logistic regression. After controlling for the effects of potentially confounding variables using multivariate logistic regression, type of street, occupation (street prostitution), and younger age became statistically significant at 0.05. ‘Off street’ types of female have a greater odds of victimization [OR (95% CI) =6.3 (3.008, 12.9)]. Being a street prostitute and age from 15–29 also increase the odds of victimization with [OR (95% CI) = 4.124(1.6, 11.1)] and [OR (95% CI) =3.533(1.1, 11.2)] respectively. See Table 3.

**Table 3:** Comparison of socio demographic and other factors with of rape among street females in Bahir- Dar, August, 2010

**Variables**	**Rape**		**COR(95% CI)**	**AOR(95% CI)**
	**No**	**Yes**		
Age(years)				
15-29**	151	79	6.7 (2.3, 19.2)	3.5(1.1, 11.2)
30-39	94	13	1.8 (.55, 5.7)	1.3(.36, 4.5)
40-49	51	4	1	1
Marital status 15-29*				
Ever married	120	22	1	1
Single*	179	74	2.5 (1.3,3.8)	1.7(.9, 3.2)
Type of street				
On the street	213	27	1	1
Off the street**	86	69	6.4 (3.8, 10.5)	6.7(3.5, 12.8)
Substance use				
Yes*	41	31	3.0 (1.8, 5.2)	1.5(.8, 2.9)
No	258	65	1	1
Disability				
No	241	63	1	1
Yes*	58	33	2.2 (1.3, 3.6)	2.2(.9,4.7)
Occupation				
Beggars	118	35	7(.4, 1.2)	.5(.3, 1.3)
Vending	106	15	3(.2, .7)	.8(.3, 1.9)
Prostitution**	16	20	2.837(1.3, 6.3)	4.1(1.5, 11.1)
No job	59	26	1	1

**Statistically significant at 95% CI.

*Their effect disappeared when adjusted for potential confounders.

## Discussion

This paper was conducted with the intention to produce a better understanding and picture on sexual violence among street female dwellers by supplementing the survey with qualitative data. Most, 75.4% of the participants had started sex with a starting median age of 17 years and 50 (16.7%) of sex initiation is attributed to rape.

Prevalence of life time rape and last one year rape was 24.3% and 11.4% respectively. The fact that rape attempt prevalence (16.2%) was less than completed rape implies that most attempts were successful by the rapist. The prevalence of rape in this study is higher than other studies [17-19]; this may be due to risky nature street life. But it is lower than the study conducted in Addis Ababa and Brazil [14,15]. This might come from the reason these studies were conducted in a street setting like this survey but both considered youths as their study subjects; it is likely that youths have a higher chance of getting raped. In addition, the higher proportion of prostitutes in those studies and their relatively high level of substance use behavior might increase the likely hood of rape in those studies.

In this study, only (6.2%) of rapes were reported to legal bodies (police and court). The result is lower than the study done in Addis Ababa but higher than the WHO multicounty survey. This report is similar to the survey done among homeless Canada’s [14,16,20]. More than half of the victims in this research didn’t report because of being ashamed. This finding is different from results from other researches [14,16,20]. This may be due to high level of illiteracy and many of our respondents came from the rural community. But the remaining replied that fear of revenge and rejection by legal bodies which are similar to above survey results. Here it is important to remind that WHO proposes health care professionals and legal bodies accept it as a major public issue and to approach it by being receptive; by recognizing women’s integrity and their entitlement to human rights; by working in conjunction with other sectors of society [21]. This was also supported by the qualitative findings where the participants reported that low awareness by the victims, fear of stigma, and fear of subsequent street life and lack of full evidence on their case as their reasons to regret female victims from reporting.

Unlike other researches which reported that most victims were attacked by the person they knew [15,20], our research showed that most rapists were not known to the victims. This finding was also supported by the female discussants who reported that most rapists were strange to them.

Pregnancy was reported as a consequence by 19% of the victims. This result was comparable with a research done among Brazilian street females, street females in Addis Ababa and students in Western shoa [14,15,19], but higher than research done in other area [22]. The difference could be explained by lower use of contraceptive methods. Vaginal discharge was reported by 22% of the participants, this is comparable to research done in other setting [23]. The percentage of induced abortion reported was also comparable to the research in Addis Ababa, however it was lower than the survey in Brazil; this may be due to the low level of knowledge about the service along with the poor availability and accessibility of this service in our setting.

Even thought we had scarce literature on the use of condom and emergency contraceptive methods during and after rape attack, our survey showed that none of the victims had used condom nor they had condom at their pocket and only 4.16% of them had used emergency contraceptive methods. Many papers agreed that women’s inability to negotiate safe sex in a violent situation increase the risk of unwanted pregnancy and sexual transmission infections [9-13]. However, it is likely that females who have condom in their pocket have a higher chance of negotiating for relatively safer sex. As complete suppression of such attacks are very difficult and need long term interventions like improving socioeconomic status, provision of condom and life skill training along with launching of emergency services for HIV/AIDS and pregnancy prevention are advisable.

Many victims claimed working during the night time and sleeping in areas where there are many brothels, and being physically weak as their reasons for being raped; this is comparable to the study in other area [14]. Similarly, both the in-depth interview and FGD participants emphasized male dominance, drinking alcohol and to work and sleep in the street as dominant explanations for the rape attack on the streets which is comparable to justifications given by male discussants among FGD undertaken in Addis Ababa. Being abused by substance and abnormal sexual orientation (behavior) are other reasons which provoke males for this act in this study.

WHO in its ecological approach model for violence against women indicated that individual factors like younger age, low woman’s level of education, financial dependency, previous victimization, low level of empowerment and lack of social support increases female risk of victimization [16,23,24]. Our study also indicated females “off” the street, being a street prostitute and age 15–29 years had a higher odds of victimization. The fact that off street females had greater risk of getting raped is a call for the government and other concerned parties to construct separate female street shelter.

Generally as we can understand from the qualitative study, particularly from the case studies, rape occurs in the mind of the rapist long before the rape occurs, and often long before the victim even meets the rapist. It’s the manipulation of the situation that the rapist uses to separate the victim from others. The crime and process seems that the issue is complex, pre-arranged and an interplay among organized people by which male dominance is reflected. The precursors lie not always in the situation. It is worthwhile that females don’t trust males who come to them during the night time. It is also valuable if females sleep far from males and drinking brothels.

This study has the following limitations; social desirability bias secondary to sensitivity of the information had been possibly encountered, temporary site and occupation change by the participants could also happen during the data collection period. Selection bias was the other limitation of the study. We were also unable to gather information from deaf women and women with intellectual disabilities.

## Conclusions

In general, this study concludes high prevalence of rape and yet many are at risk of it. Many of rapes were left unreported to legal bodies and prevention practices for protecting health thereafter the rape event was very low. Street female victims were suffering from unwanted pregnancy, symptoms of STDs and other psychological consequences. Sleeping in areas where there are many brothels, working and sleeping with males, being physically weak, long stay in the street, male dominance, substance abuse and abnormal sexual orientation by males were the reasons which increased female’s victimization by rape. Females “off” the street, being a street prostitute and age 15–29 were significantly associated with increased risk of rape.

## Competing interests

The authors declare that they have no competing interests.

## Authors’ contributions

MCA involved much in conception, design and acquisition, analysis and interpretation of data as well as he carried out drafting the manuscript. WAY participated in revising the manuscript critically for important intellectual contents and has also participated in developing the final manuscript. Both authors read and approved the final manuscript.

## Authors’ information

Worku A Yalew is the co-author.

## Pre-publication history

The pre-publication history for this paper can be accessed here:

http://www.biomedcentral.com/1471-2458/13/825/prepub
